# Health-Related Effects of Genetic Variations of Alcohol-Metabolizing Enzymes in African Americans

**Published:** 2007

**Authors:** Denise M. Scott, Robert E. Taylor

**Keywords:** Alcohol and other drug (AOD) use, abuse, and dependence, drinking behavior, African American, ethanol metabolism, alcohol dehydrogenase (ADH), aldehyde dehydrogenase (ALDH), acetaldehyde, genetic factors, genetic polymorphisms, allele, ADH1B, ADH1B*3, ALDH1A1*2, ALDH1A1*3, protective factors, alcohol flush reaction

## Abstract

Alcohol metabolism involves two key enzymes—alcohol dehydrogenase (ADH) and aldehyde dehydrogenase (ALDH). There are several types of ADH and ALDH, each of which may exist in several variants (i.e., isoforms) that differ in their ability to break down alcohol and its toxic metabolite acetaldehyde. The isoforms are encoded by different gene variants (i.e., alleles) whose distribution among ethnic groups differs. One variant of ADH is ADH1B, which is encoded by several alleles. An allele called ADH1B*3 is unique to people of African descent and certain Native American tribes. This allele is associated with more rapid breakdown of alcohol, leading to a transient accumulation of acetaldehyde. African Americans carrying this allele are less likely to have a family history of alcoholism and experience a less rewarding subjective response to alcohol. Moreover, children of mothers with this allele are less vulnerable to alcohol-related birth defects. The enzyme ALDH1 also is encoded by several alleles. Two of these alleles that are found in African Americans—ALDH1A1*2 and ALDH1A1*3—may be associated with a reduced risk of alcoholism.

Alcohol metabolism is one of the biological determinants that can influence drinking behavior and the development of alcohol dependence and alcohol-induced organ damage (Yin and Agarwal 2001). Oxidative alcohol metabolism depends on two key enzymes— alcohol dehydrogenase (ADH) and aldehyde dehydrogenase (ALDH). ADH converts alcohol to the highly toxic metabolite acetaldehyde, which then is metabolized by ALDH to acetate and eventually to carbon dioxide (CO_2_) and water. For both ADH and ALDH, several variants (i.e., isoforms) exist that differ in their ability to break down alcohol and acetaldehyde, respectively. For example, certain ADH isoforms are particularly active and rapidly break down alcohol to acetaldehyde; conversely, certain ALDH isoforms have very low activity and break down acetaldehyde slowly. In both of these cases, acetaldehyde will accumulate after alcohol consumption, exerting its toxic effects (e.g., causing a flushing syndrome characterized by facial flushing, nausea, and rapid heartbeat.) These effects deter people from alcohol consumption and therefore have a protective effect against alcoholism.

Because alcohol metabolism can significantly influence drinking behavior and the risk for alcoholism (Yin and Agarwal 2001), it is important to understand how genetic factors influence metabolism. The different ADH and ALDH isoforms are encoded by different genes, each of which again may be present in different variations (i.e., alleles). The occurrence of alternate alleles in a population is termed polymorphism. The activity and distribution of the *ADH* and *ALDH* alleles and of the proteins they encode have been intensively studied. For example, researchers have identified differences across ethnic groups in the frequencies with which individual genes or gene variants occur (Osier et al. 2002; Chou et al. 1999). This brief article examines the prevalence and effects of genetic variants of *ADH* and *ALDH* genes in African Americans.

## ADH Polymorphisms in African Americans

There are numerous types of ADH, which, based on their structural similarities, can be categorized into five classes. (For more information on the classification and subtypes of ADH and ALDH enzymes, see the article by Edenberg.) The class I ADH enzymes include ADH1A, ADH1B, and ADH1C, which are considered the most important ADH isoforms in alcohol metabolism because they are present in the largest amounts and account for the majority of alcohol metabolism in humans (Jornvall and Hoog 1995). The genes encoding the class I ADH enzymes are called *ADH1A*, *ADH1B*, and *ADH1C*. For some of these genes, several alleles have been identified, particularly for the *ADH1B* gene. For this gene, there are three main alleles— ADH1B*1, ADH1B*2, and ADH1B*3—that result in significant differences in alcohol metabolism.

The prevalence of the alleles varies in different ethnic populations. ADH1B*1 is the most common allele and occurs in varying frequencies in all populations. The ADH1B*2 allele is present in the majority of Far East Asians (Goedde et al. 1997) and in smaller percentages of Caucasians (Borras et al. 2000), people of Jewish descent (Shea et al. 2001; Hasin et al. 2002), and African Americans (Luo et al. 2006). Finally, ADH1B*3 has been found in people of African descent and certain tribes of Native Americans (Thomasson et al. 1995; Ehlers et al. 2001, 2003).

**At a Glance**

The ADH1B*3 Allele

- The ADH1B*3 allele has been found in up to one-fourth of people of African descent (Bosron and Li 1987; Luo et al. 2006).
- It is associated with a high alcohol elimination rate, intense response to alcohol, and decreased risk of alcohol-related birth defects (Thomasson et al. 1995; Ehlers et al. 2003; McCarver-May et al. 1997).
- Evidence suggests that the allele is associated with a protective effect against alcoholism (Ehlers et al. 2001; Edenberg et al. 2006; Luo et al. 2006).

Research into the genetics of alcohol metabolism in the African-American population is very limited; however, several studies, which are reviewed below, have examined important aspects of alcohol use that may be linked to the ADH1B*3 allele.

### ADH1B*3 and Family History of Alcoholism

The ADH1B*3 allele occurs in approximately 15 to 25 percent of African Americans (Bosron and Li 1987; Luo et al 2006). In another study of 97 African Americans, 31 percent had at least one ADH1B*3 allele and 2 percent had two ADH1B*3 alleles (i.e., were homozygous for the allele) (Ehlers et al. 2001).^1^ In that study, participants with at least one ADH1B*3 allele were significantly less likely to have a family history of alcohol dependence than those without the allele. Because family history of alcohol dependence is one of the best predictors of alcohol abuse and alcohol dependence, the investigators concluded that the ADH1B*3 allele may be associated with a lowered risk for alcohol dependence (Ehlers et al. 2001).^2^ This conclusion is supported by other recent studies, which also found that the ADH1B*3 allele was significantly associated with a reduced risk for alcohol dependence among African Americans (Edenberg et al. 2006; Luo et al. 2006).

### ADH1B*3 and Alcohol-Related Expectations

The mechanism by which the presence of certain alleles may affect a person’s drinking behavior is unclear; however, numerous studies (Brown et al. 1980; Christiansen and Goldman 1983; Leigh 1989; Rohsenow 1983; Smith et al. 1995) have demonstrated that expectations about the consequences of alcohol consumption are related to drinking behavior in adolescents and adults. Little is known about alcohol expectations in African Americans, but one study (Ehlers et al. 2003) examined the association between alcohol-related expectations and the presence of the ADH1B*3 allele in 66 African Americans. The alcohol-related expectations evaluated in this study assessed positive personal beliefs, feelings, and experiences associated with alcohol consumption, such as enhanced sexual performance, physical/social pleasure, and increased assertiveness. The researchers concluded that the presence of the ADH1B*3 allele may be associated with a less rewarding subjective response to alcohol, an effect that has been associated with protection from the development of alcohol use disorders in other populations.

### ADH1B*3 and Alcohol Elimination Rates

Alcohol’s pharmacological effects depend on how much a person drinks (i.e., the blood alcohol levels achieved) and how long the alcohol stays in his or her system before it is metabolized. It has been suggested that the ADH1B*3 allele may encode a highly active ADH enzyme that leads to more efficient alcohol metabolism, resulting in more rapid lowering of blood alcohol levels and transient accumulation of acetaldehyde (Crabb 1995). Because acetaldehyde induces unpleasant or toxic effects, people carrying alleles of *ADH1B* and other alcohol-metabolizing enzymes that lead to acetaldehyde accumulation may consume less alcohol to avoid acetaldehyde’s effects and may therefore be less likely to develop alcohol dependence. To test the hypothesis that ADH1B*3 is associated with more rapid alcohol elimination and acetaldehyde accumulation, Thomasson and colleagues (1995) conducted a study that evaluated alcohol elimination rates and *ADH1B* polymorphisms in 326 African Americans. The researchers found that participants with an ADH1B*3 allele had significantly faster alcohol elimination rates compared with participants who carried only the ADH1B*1 allele. This finding suggests that the ADH1B*3 allele is associated with a higher rate of alcohol metabolism and supports the idea that the allele encodes a functional enzyme that has a protective effect against alcoholism.

### ADH1B*3 and Vulnerability to Alcohol-Related Birth Defects

Research with African-American mothers and infants suggests that the presence of the ADH1B*3 allele in the mother may reduce the risk of alcohol-induced fetal/infant impairment. McCarver-May and colleagues (1996) found that among African-American women who consumed an average of more than one drink per day (i.e., more than 0.5 ounces of pure alcohol), those with an ADH1B*3 allele were less likely than those without the allele to have infants with characteristics of fetal alcohol spectrum disorder.^3^ These findings propose that the higher alcohol elimination rate in people with the ADH1B*3 allele has a protective effect against alcohol-induced developmental changes, suggesting that the fetus is exposed for a shorter period of time to any alcohol the mother may have consumed.

In a subsequent study, the researchers followed 243 African-American mother–infant pairs, evaluating the mothers’ *ADH1B* status and alcohol use during pregnancy as well as the infants’ performance on tests of mental development at 12 months of age (McCarver et al. 1997). The study found that African-American women with the ADH1B*3 allele were less likely to bear children with alcohol-related birth defects than women without the allele. Only children whose mothers consumed alcohol during the pregnancy and did not have the ADH1B*3 allele showed lower scores on tests of mental development. These results confirm the findings of the previous study, and the researchers conclude that the ADH1B*3 allele may be associated with more efficient alcohol metabolism even at high blood alcohol levels. Moreover, the investigators suggest that these observations support the hypothesis that it is alcohol rather than acetaldehyde that induces fetal defects (i.e., acts as a teratogen) (McCarver et al. 1997). Thus, these studies suggest that a specific genetic predisposition (i.e., presence or absence of the ADH1B*3 allele) influences the susceptibility to alcohol-related birth defects.

### ALDH Polymorphisms in African Americans

In addition to the ADH polymorphisms discussed above, research also has found important links between the risk of alcoholism and certain ALDH polymorphisms in African Americans. There are two main types of ALDH— ALDH1 and ALDH2. ALDH1, which is encoded by the *ALDH1A1* gene, has been associated with alcoholism, alcohol-induced flushing, and alcohol sensitivity (Chan 1986; Yoshida 1992). Three alleles of the *ALDH1A1* gene (ALDH1A1*1, ALDH1A1*2, and ALDH1A1*3) have been identified. In one recent study (Spence et al. 2003) involving a variety of ethnic groups, including African Americans, the ALDH1A1*2 allele was found with a low frequency in several ethnic groups, whereas the ALDH1A1*3 allele was found only in African Americans. Moreover, the results of that study suggested that the ALDH1A1*2 and ALDH1A1*3 alleles may be associated with a reduced risk of alcoholism. The researchers noted, however, that further research is needed to validate this observation.

## Conclusions and Future Research Needs

Several *ADH* and *ALDH* alleles have been identified primarily or exclusively in African Americans and appear to offer some protection from the risk of alcoholism. For example, the ADH1B*3 allele, which has been found in up to one-fourth of people of African descent studied and which results in a higher rate of alcohol metabolism, was associated with a reduced likelihood of a family history of alcoholism, less positive response to alcohol, and protection against alcohol-related birth defects. Similarly, the ALDH1A1*3 allele, which has been identified exclusively in African Americans, also may be associated with a reduced risk of alcoholism. However, further research is needed to identify additional allelic variations of the genes encoding ADH and ALDH in African Americans and to examine in more detail how all of these variations affect enzyme function and what impact these effects have on drinking behavior and risk of alcoholism. Investigations into the mechanisms that control ADH and ALDH activities, as well as research to accurately measure the rates at which alcohol and its first metabolite acetaldehyde are eliminated, also are of great importance. Together, the results of these studies likely will improve our understanding of the relative risks of alcohol dependence and health consequences for people of all ethnicities who drink alcoholic beverages, including African Americans.

## Footnotes

1 Every person carries two copies of each gene, one inherited from the father and one inherited from the mother. If both copies are of the same allele, the person is called homozygous for that allele. If the two copies are of different alleles, the person is called heterozygous.

2 The researchers did not find any significant associations between the presence of an *ADH1B*3* allele and a personal history of alcohol abuse or dependence or current drinking. However, the ability to detect associations was limited because of the small sample size and because half of the participants did not drink regularly.

3 Fetal alcohol spectrum disorder includes fetal alcohol syndrome (FAS), fetal alcohol effects (FAE), alcohol-related birth defects (ARBD), and alcohol-related neurodevelopmental disorder (ARND) (for a review, see Sampson et al. 1997). Characteristics of these disorders include, among others, lower birth weight, smaller head circumference, and lower scores on the Mental Developmental Index (MDI) of Bayley Scales of Infant Development Mental Index (BSID).
